# Understanding Fear of Opportunism in Global Prize-Based Science Contests: Evidence for Gender and Age Differences

**DOI:** 10.1371/journal.pone.0134898

**Published:** 2015-07-31

**Authors:** Oguz Ali Acar, Jan van den Ende

**Affiliations:** 1 Department of Management, King’s College London, London, United Kingdom; 2 Department of Technology and Operations Management, Rotterdam School of Management Erasmus University, Rotterdam, Netherlands; Mälardalen University, SWEDEN

## Abstract

Global prize-based science contests have great potential for tapping into diverse knowledge on a global scale and overcoming important scientific challenges. A necessary step for knowledge to be utilized in these contests is for that knowledge to be disclosed. Knowledge disclosure, however, is paradoxical in nature: in order for the value of knowledge to be assessed, inventors must disclose their knowledge, but then the person who receives that knowledge does so at no cost and may use it opportunistically. This risk of potential opportunistic behavior in turn makes the inventor fearful of disclosing knowledge, and this is a major psychological barrier to knowledge disclosure. In this project, we investigated this fear of opportunism in global prize-based science contests by surveying 630 contest participants in the InnoCentive online platform for science contests. We found that participants in these science contests experience fear of opportunism to varying degrees, and that women and older participants have significantly less fear of disclosing their scientific knowledge. Our findings highlight the importance of taking differences in such fears into account when designing global prize-based contests so that the potential of the contests for reaching solutions to important and challenging problems can be used more effectively.

## Introduction

The internet and advances in communication technologies have provided unprecedented opportunities for accessing and incorporating diverse knowledge all over the world. Researchers have emphasized the importance of creating a global pool of knowledge that can be used to overcome the overwhelming challenges humanity faces in the 21^st^ century, such as developing energy sources that do not contribute to climate warming [1–3]. One effective means of bringing together scientific knowledge from across the world are online prize-based science contests that are global in scope. These contests have remarkable potential in solving challenging scientific problems in various disciplines, such as breakthroughs in the discovery and development of new drugs or ways of dealing with the challenges of big data in the biological sciences [4–6].

Knowledge disclosure is a necessary condition for the utilization of knowledge in global prize-based science contests (hereafter referred to as prize contests) [7,8]. In this respect, Nobel laureate economist Kenneth Arrow highlights the paradoxical nature of knowledge disclosure: the value of knowledge can only be determined after one has the knowledge, but then the receiver of the knowledge acquires it without any cost [9]. The purchaser then might act opportunistically and misappropriate the information, which will in turn make the inventor fearful of disclosing knowledge. This fear of opportunism is a major barrier to disclosure of knowledge [9,10]. However, our understanding of fear of opportunism in prize contests is limited. Although prior research has extended our understanding by focusing on the role of expertise, competition and incentives in prize contests [11–14], to the best of our knowledge, no prior empirical study has focused on participants’ fear of opportunism in such contests. A better understanding of, and a specific focus on, the fears of prize contest participants in relation to knowledge disclosure is of great importance because the knowledge created and disclosed by this group of people has great potential in overcoming important scientific challenges [4–6,15]

In this project, our main aim is to contribute to a deeper and more fine-grained understanding of the fear of opportunism among the individuals who actively participate in prize contests. More specifically, we question whether the people who generate and disclose solutions in these contests are a specific population of individuals who do not experience fear of opportunism when disclosing their knowledge or whether they disclose their knowledge *despite* experiencing fear of opportunism. In addition, we explore whether these fears are the same among different individuals and demographic groups. Our study therefore contributes to the literature on prize contests and knowledge disclosure in two ways. First, we take the first steps toward understanding the fear of opportunistic behavior in the emerging context of online prize contests. Second, we identify demographic differences in how contest participants experience their fear of opportunism. Our findings thereby question the previous economics research on knowledge disclosure which has often considered fear of opportunism to be uniform among different individuals.

### Overview of the present research

In an attempt to understand fear of opportunism in prize contests, we conducted a survey with those contributing solutions on the InnoCentive online platform. InnoCentive applies crowdsourcing principles in the scientific realm by broadcasting scientific prize-based contests online and awarding financial prizes for the best solutions, ranging from 5,000 to 1,000,000 USD [16]. InnoCentive is one of the best-known examples of how organizations can tap into a global knowledge pool and how people from all over the world and various scientific disciplines can solve challenging scientific problems. Researchers and science writers often highlight the potential of InnoCentive contests to overcome the scientific challenges we encounter [4,6,15,16].

## Methods

### Ethics statement

Potential participants were invited via an email which described the purpose, procedure and anticipated benefits of the research. The email also explained institutional affiliations of the researchers, anonymity of the responses and that the data will only be used for academic purposes. Participation to the survey was completely voluntary and the respondents did not have any dependencies to the researchers. Survey data were collected only from those who agreed to proceed by clicking the survey link in the email. This voluntary agreement and electronic submission of the completed questionnaire implied consent. Written or verbal consent was not sought as the survey was answered online and anonymously. The study, survey and consent procedure were approved by the Institutional Review Board of the Rotterdam School of Management, Erasmus University. On a related note, a confidentiality agreement, which protects the privacy and rights of the participants, was signed between the researchers and InnoCentive.

### Participants, design and procedure

We conducted our study in collaboration with InnoCentive, which offers an online platform for prize-based science contests. This platform matches scientific problems of its clients (i.e. seekers) to its community (i.e., solvers). Seekers share a specific problem—that typically could not be solved by the internal R&D department of the company—with InnoCentive, and InnoCentive then broadcasts this problem on its online platform in the form of a contest. After a challenge is posted, solvers submit their ideas and solutions by means of a written report. The winning solution is selected by the seeker and rewarded with a prize. Given that InnoCentive platform consists of wide range of problems that are solved in a contest format and that InnoCentive community consists of people from all over the world (i.e., nearly 200 countries), we believe it represents an appropriate platform for studying global prize-based science contests. It is important to note two points about the knowledge disclosure in InnoCentive. First, to get legal ownership of a solution, organizations must give the prize money to owner of the solution. That is, organizations get to see the solutions for free but do not get the legal ownership of the solutions for free. Second, there is no evidence that seekers actually engage in opportunistic behavior (e.g., steal information from those who propose the solutions).

We focused on two specific types of contests that are broadcasted in the InnoCentive platform, namely Reduction-to-Practice (RTP) and Theoretical contests. RTP contests require a detailed description of the solution and physical evidence, which proves that the solution will work. Theoretical contests require a detailed description of the solution and supporting precedents of the solution. Examples of RTP and Theoretical contests are development of “an enzyme stabilizer at high pH” and formulation of “a simple, stable, and safe injectable suspension placebo that has no pharmacological and biological activity”, respectively [12]. The reason why we focus on these contests is that both types of contests seek solutions to scientific problems (from different scientific disciplines such as chemistry, biology or physics) and, therefore, are more suitable for our research questions. Focusing on these contests was important to remain focused on scientific problem solving because InnoCentive also had contests that seek solutions to non-scientific problems such as business model or marketing-related problems. Our sample consisted of all solvers (i.e., 3005 solvers) who had participated in at least one RTP or Theoretical contest in 2.5 years prior to the data collection (i.e., between December 2009 and May 2012). As we reached out to all solvers who had participated to prize-based contests that address a scientific problem over a relatively large timespan, we expect our sample to be representative of active participants of prize-based science contests in the InnoCentive platform.

We used a web-based survey tool to collect data from the RTP and Theoretical contest participants in the InnoCentive online platform. The entire survey was in English as the contest information in InnoCentive was broadcasted in English. In total, using contact information retrieved from InnoCentive, we sent a customized email including a URL link to our survey, to 3005 contest participants to invite them to participate in the survey. The e-mail made plain that the researchers were partnering with InnoCentive and that solvers’ responses were very important to us. Solvers were also informed by a manager from InnoCentive, via the LinkedIn groups and the InnoCentive blog, that InnoCentive was collaborating with us and that solvers might soon receive an email about the study. A reminder was sent a week after the initial contact, using a dynamic strategy (i.e., the time, day and text of the initial email was changed) to enhance the response rate [17]. One week after the reminder the survey was closed. We received 744 (24.8%) responses, of which 630 (21.0%) were usable for further analyses (i.e., had answers for at least one construct of this study). We decided to keep the responses when questions for one or more constructs of our study were answered because we wanted to utilize the data that is usable in pairwise analyses of the constructs. The findings that are reported in the next section remained similar when we excluded the responses that are not entirely complete (i.e., cases that did not answer questions for one or more constructs of this study).

To assess whether the non-response bias was an important issue for our study, we compared the answers of early respondents (i.e., those who responded before the reminder) and late respondents (i.e., those who responded after the reminder). The assumption in this analysis is that late respondents are closer to the non-responding group than the early respondents [18]. Independent sample *t*-test showed no significant differences between early and late respondents in any of the variables measured in this study, namely fear of opportunism, gender, age, education and income level (p = 0.41; 0.44; 0.87; 0.84; 0.14 respectively). We also conducted the same analysis for the first 10% of the respondents and the last 10% of the respondents. The comparison of these two groups also did not reveal any significant differences for fear of opportunism, gender, age, education and income level variables (p = 0.35; 0.18; 0.31; 0.82; 0.42 respectively). In addition, a previous survey conducted with InnoCentive contest participants (i.e., solvers who have participated in at least one RTP or Theoretical contest) reported that survey respondents did not have statistically significant differences from non-responders in demographic characteristics such as gender distribution and ethnicity [12]. The percentage of female solvers reported in the same study for survey respondents and non-respondents (10% and 11%, respectively) is comparable to the percentage of female in our sample (9%). Taken together, although it cannot be ruled out completely with the data we have, we do not expect non-response bias to be a major concern for our study.

In the survey, we asked participants to fill information on four demographic variables: age, gender, education and income level. We measured age in years, and it was reported in an open-ended question in our survey. Gender was measured by asking respondents to indicate whether they were male or female. Education level was assessed by the highest academic degree earned and had 6 levels ranging from “less than a high school degree” to “PhD degree”. For the income level variable, respondents were asked to report their annual income as within one of 8 ranges. The lowest income level was “0 to 25,000 USD” and the highest one was “more than 500,000 USD”.

To measure the latent construct of fear of opportunism, we adapted a validated scale from prior research to our context [19]. The original scale was designed to address the opportunism in an inter-firm context and for the relationships between an organization and its’ partners (e.g., suppliers). Morgan and Hunt [19] used a shorter version (3 items) of the 6-item opportunism scale developed by John [20]. The items of the original scale were developed on the basis of the conceptual definition of the opportunism construct, an exploratory field interview and an observation study. After developing a set of items for the scale, 5 expert judges assessed face validity of the scale items and items that were considered as unsuitable by at least one of the expert judges were excluded. Following this step, the survey was sent to a sample of organizations. On the basis of the answers to the questionnaire, some other unsuitable items (i.e., items with an item-total correlation value that is less than 0.3) were deleted. The final opportunism scale had a one-factor structure. To verify single-factor structure eigenvalue rule and a chi square statistic were used [20].

We modified this scale in such a way that it explicitly addresses the relationship between a contest participant and contest beneficiary (i.e., seeker) and specific potential opportunistic behaviors in this relationship. For modifying the scale, we used the information we gathered from 23 in-depth interviews that we conducted with contest participants in InnoCentive and employees of InnoCentive. More specifically, we interviewed contest participants who had won prizes multiple times, had won a prize once and had not won any prizes. Employees that we interviewed had extensive knowledge about contest participants and were from different organizational departments. All interviews were tape-recorded. In the interviews we asked interviewees to explain their relationship with seekers and InnoCentive, and identify potential opportunistic behaviors they fear. The results revealed that solvers mainly fear about potential usage of their solutions by seeker without receiving promised award(s). In the light of this information, for example, an item focusing on not keeping promises was modified in such a way that it addresses not keeping the main promise in our context—using a solution without paying the solver who developed it. We assessed fear of opportunism with three items: “I think seekers will steal my ideas”, “I think seekers will change the facts in order not to pay me the award I deserve”, “I think seekers will use my solution without paying me”. In the scale, we used 7-point scale anchors measuring the extent of agreement which ranged from “totally disagree” to “totally agree”. If an item of the scale was not answered, we used remaining answered items to create an average score for fear of opportunism.

The internal consistency of the fear of opportunism scale was high, with a Cronbach’s α value of 0.88. To examine factorial validity of our fear of opportunism scale, we first conducted a factor analysis using principal components extraction method, and Eigenvalue (i.e., extracting factors that have an Eigenvalue greater than 1) and scree plot criteria. The analysis suggested that a single-factor solution fitted best with our data. This solution explained 80.6% of variance (Eigenvalue = 2.42). Factor loadings of the items were quite high, namely 0.88, 0.92 and 0.89.

In addition, we examined nomological validity of our scale by investigating the correlation between fear of opportunism and trust. Prior research identified trust as a main factor in mitigating opportunism and consistently found a negative association between them [19,21]. Our data confirmed this expectation as the correlation between trust to InnoCentive and fear of opportunism was negative and significant (r = -0.55, p < 0.001). We measured trust with seven items (*α* = .87). The items were adapted from an established and widely used trust scale [22]. The original scale was designed to measure trust between an employer and employee. We adapted the items in such a way that “my employer” statement in the original items was replaced by “InnoCentive”. The items used were: “I believe InnoCentive has high integrity”, “I can expect InnoCentive to treat me in a consistent and predictable fashion”, “InnoCentive is not always honest and truthful”, “In general, I believe InnoCentive’s motives and intentions are good”, “I don’t think InnoCentive treats me fairly”, “InnoCentive is open and upfront with me”, I am not sure if I fully trust InnoCentive”. In order to assess discriminant validity of trust and fear of opportunism, we conducted confirmatory factor analysis using LISREL software. Expected 2-factor solution provided a good fit with the data (*χ* ^*2*^ = 158.90, RMSEA = .09, SRMR = .05, CFI = .98, GFI = .95) and a better fit than alternative one factor solution (*χ* ^*2*^ = 446.71, RMSEA = .16, SRMR = .08, CFI = .93, GFI = .87). Chi square difference tests also showed that our expected model has a significantly better fit than one factor solution, which suggests that trust and fear of opportunism constructs are distinct.

## Results

**Fig 1:**
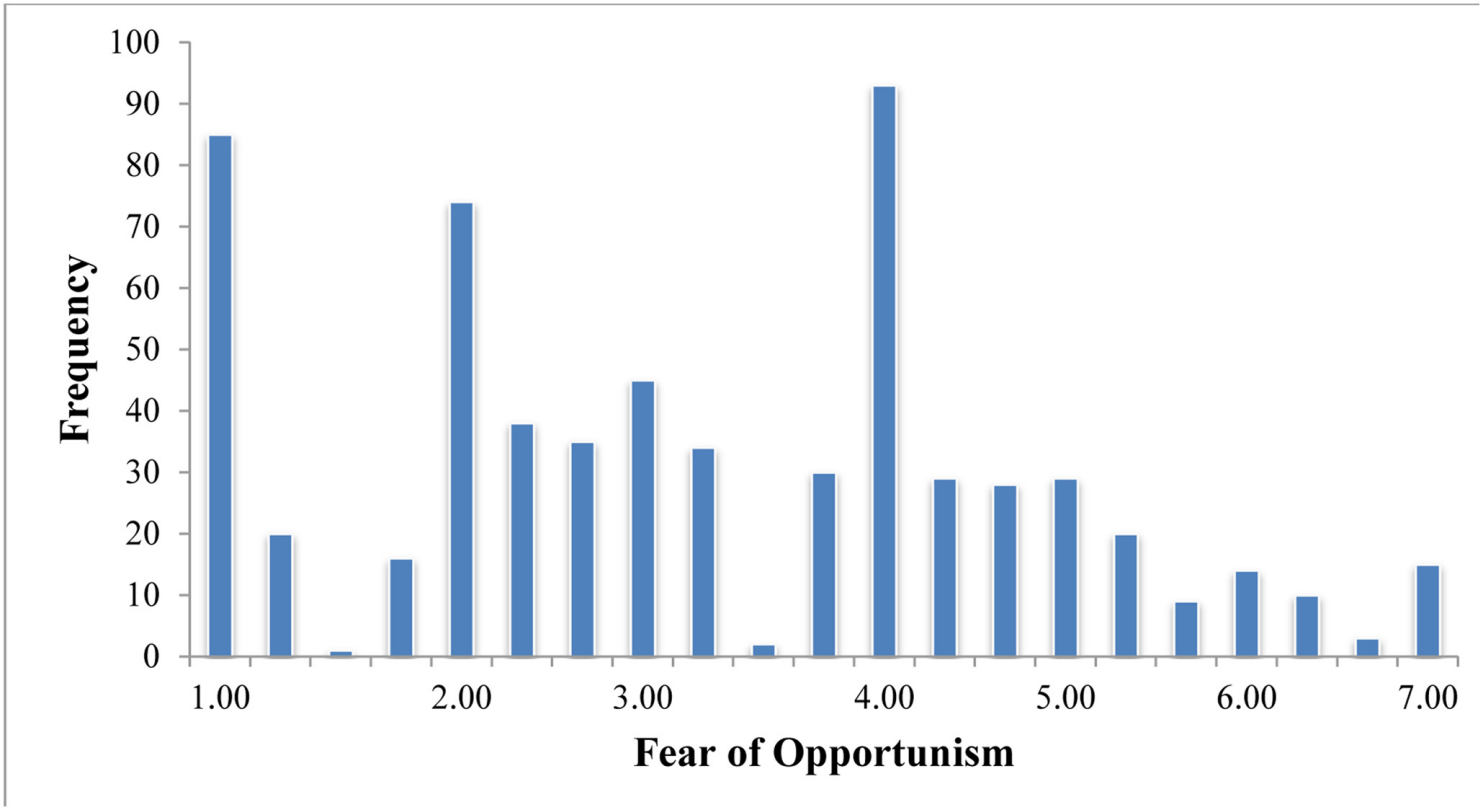
Histogram of responses to fear of opportunism scale.

**Fig 2:**
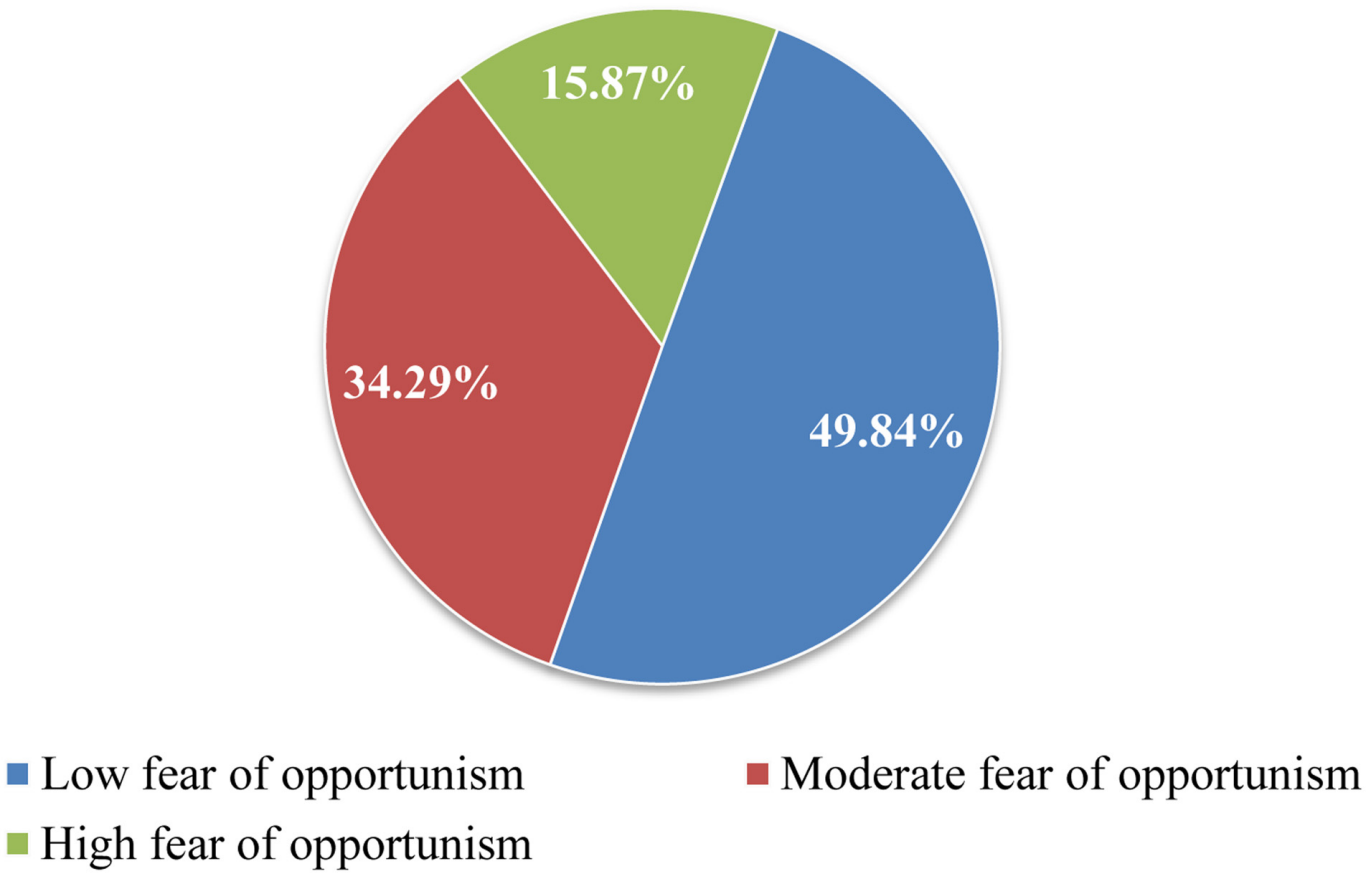
Pie chart illustrating proportion of fear of opportunism levels among contest participants. Respondents in the low, moderate and high fear of opportunism groups consisted of participants that had an average rating within the ranges of “3 or less”, “from 3 to 5” and “5 or more” in the fear of opportunism scale, respectively.

The average age of our sample was 44.29 (*SD* = 14.80), 91% of our sample was male, and 81.6% of the sample had at least an undergraduate degree. Table 1 presents detailed sample characteristics and self-reported fear of opportunism score for each category of gender, age and income level variables. We started our analyses by exploring how fear of opportunism was experienced among contest participants. On average, contest participants experienced moderate levels of fear of opportunism (*M* = 3.24) and they experienced such fears to varying degrees (*SD* = 1.56). This variation is depicted in more detail in Fig 1 which displays the histogram of responses to the fear of opportunism scale. As shown in the figure, some respondents participated in contests despite experiencing high fear of opportunism whereas other contest participants experienced low to moderate levels of fear of opportunism. To facilitate the interpretation of the distribution of fear of opportunism among contest participants, we created a pie chart that presents the proportion of respondents that experienced low, moderate and high fear of opportunism levels compared to whole sample (See Fig 2). Groups were defined based on respondents’ answers to the items of fear of opportunism scale. Respondents in the low, moderate and high fear of opportunism groups consisted of participants that had an average rating within the ranges of “3 or less”, “from 3 to 5” and “5 or more” in the fear of opportunism scale, respectively. The chart shows that a relatively low number of respondents (i.e., 15.87% of all respondents) participated in prize contests in spite of experiencing high fear of opportunism. Almost half of the sample (i.e., 49.84% of all respondents) was not particularly concerned about opportunistic behavior (i.e., had low fear of opportunism) whereas the remaining group (i.e., 34.29% of all respondents) had moderate levels of fear of opportunism.

**Table 1:** Sample characteristics and average fear of opportunism score for different categories of gender, education and income level variables.

Variable	Category	*N (%)*	*M*	*SD*
Gender	Female	56 (9%)	2.87	1.56
	Male	568 (91%)	3.26	1.56
Education level	Less than high school degree	8 (1%)	2.63	1.00
	High school degree or equivalent	41 (7%)	3.46	1.65
	Associate degree or equivalent	61(10%)	3.20	1.64
	Bachelor’s degree	147 (23%)	3.30	1.56
	Master’s degree	158 (25%)	3.12	1.42
	PhD degree	209 (33%)	3.24	1.65
Income Level	Less than $25,000	233 (39%)	3.34	1.56
	$25,000 to $49,999	115 (19%)	3.23	1.49
	$50,000 to $74,999	99 (17%)	3.02	1.41
	$75,000 to $99,999	64 (11%)	3.23	1.68
	$100,000 to $149,999	52 (9%)	3.28	1.57
	$150,000 to $249,999	21(4%)	3.43	1.73
	$250,000 or $499,999	9 (2%)	2.74	2.15
	$500,000 or more	3 (1%)	2.56	1.07

To assess the associations between demographic variables and fear of opportunism, we conducted ordinary least squares regression analyses. Table 2 presents the results of the three regression analyses that we performed. In the first analysis (i.e., Model 1), we treated income and education level variables as continuous variables. Education level scale was treated as a 6-point scale (*M* = 4.66, *SD* = 1.28, min = 1, max = 6) and income level scale was treated as an 8-point scale (*M* = 2.50, *SD* = 1.63, min = 1, max = 8). In the second analysis (i.e., Model 2), we conducted a regression analysis with different operationalization of education and income level variables. Specifically, we treated education and income level variables as categorical variables; we created 5 and 7 dummy variables to measure education and income level categories, respectively. In the third analysis (i.e., Model 3), we excluded the education and income level categories that had less than 50 respondents from the regression analysis. As a whole, these three regression analyses returned parallel results which suggested that neither the small sample size in some categories of education and income level variables nor the potential alternative operationalizations of these variables affected our results and conclusions in general.

**Table 2:** Ordinary least squares regression analysis for fear of opportunism.

Variables	Model 1^a^		Model 2^b^		Model 3^c^	
	*β*	*SE*	*β*	*SE*	*β*	*SE*
Gender	**0.09** *	0.23	**0.09** *	0.23	**0.09** *	0.24
Age	**-0.15** **	0.01	**-0.15** **	0.00	**-0.11** *	0.00
Education level	0.02	0.05				
Income level	-0.01	0.04				
High school degree			0.04	0.49		
Associate degree			0.02	0.47		
Bachelor’s degree			0.06	0.45	0.01	0.24
Master’s degree			0.01	0.44	-0.03	0.24
PhD degree			0.06	0.44	0.01	0.23
Income (25-50K)			-0.01	0.18	-0.04	0.19
Income (50-75K)			-0.05	0.19	-0.08	0.20
Income (75-100K)			0.00	0.23	-0.02	0.23
Income (100-150K)			0.02	0.25	0.00	0.26
Income (150-250K)			0.03	0.37		
Income (250-500K)			-0.04	0.54		
Income (500K and more)			-0.03	0.92		

*Note*: Gender is dummy coded: Female = 0, Male = 1. Age was measured in years. Education level variable had 6 levels ranging from “less than a high school degree” to “PhD degree”. Income level variable had 8 levels ranging from “0 to 25,000 USD” to “more than 500,000 USD”. Fear of opportunism was self-reported on a 7-point scale.

^a^ R^2^ = 0.03, F_4,581_ = 3.94, p < 0.01. Education and income level variables were treated as continuous variables measured by 6-point and 8-point scales, respectively.

^b^ R^2^ = 0.04, F_14, 595_ = 1.51, p = 0.10. Education and income level variables were treated as categorical variables. For the education and income level variables, 5 and 7 dummy variables were created, respectively.

^c^ R^2^ = 0.03, F_9, 521_ = 1.58, p = 0.12. Education and income level categories that had less than 50 respondents were excluded from the analysis (in total 82 cases). That is, “less than a high school degree” and “high school degree or equivalent” categories for the education level variable, and “150K to 250K”, “250K-500K” and “more than 500K” categories for the income level variable were dropped from the analysis.

* p < 0.05

** p < 0.001

**Fig 3:**
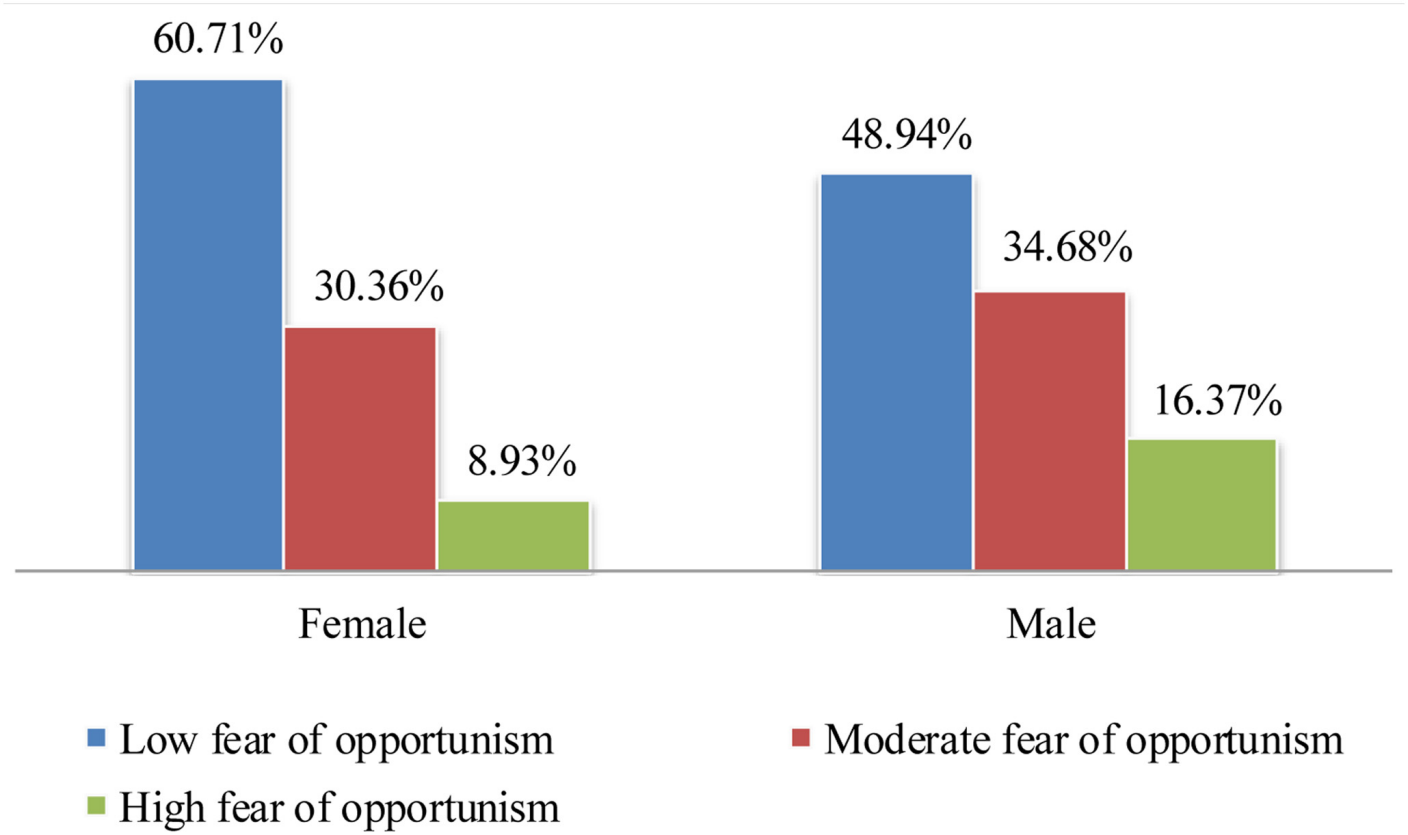
Bar chart illustrating proportion of fear of opportunism levels among male and female contest participants. Respondents in the low, moderate and high fear of opportunism groups consisted of participants that had an average rating within the ranges of “3 or less”, “from 3 to 5” and “5 or more” in the fear of opportunism scale, respectively.

In all of the analyses, association between gender and fear of opportunism was positive and statistically significant (in Model 1 β = 0.09, p < 0.05; in Model 2 β = 0.09, p < 0.05; in Model 3 β = 0.09, p < 0.05) which suggested that men experienced more fear of opportunism than women in prize contests. To examine this gender difference further, we created a bar chart that displays how the proportion of respondents that experience low, moderate and high levels of fear of opportunism varies for the male and female samples (see Fig 3). The same procedure above was followed for defining low, moderate and high fear of opportunism groups. As shown in Fig 3, the proportion of participants that experience low fear of opportunism in the female sample is higher than the male sample (i.e., 60.71% of the female respondents versus 49.84% of the male respondents). On the contrary, relatively larger proportion of male participants experienced high fear of opportunism compared to women participants (i.e., 16.37% of the male respondents versus 8.93% of the female respondents).

**Fig 4:**
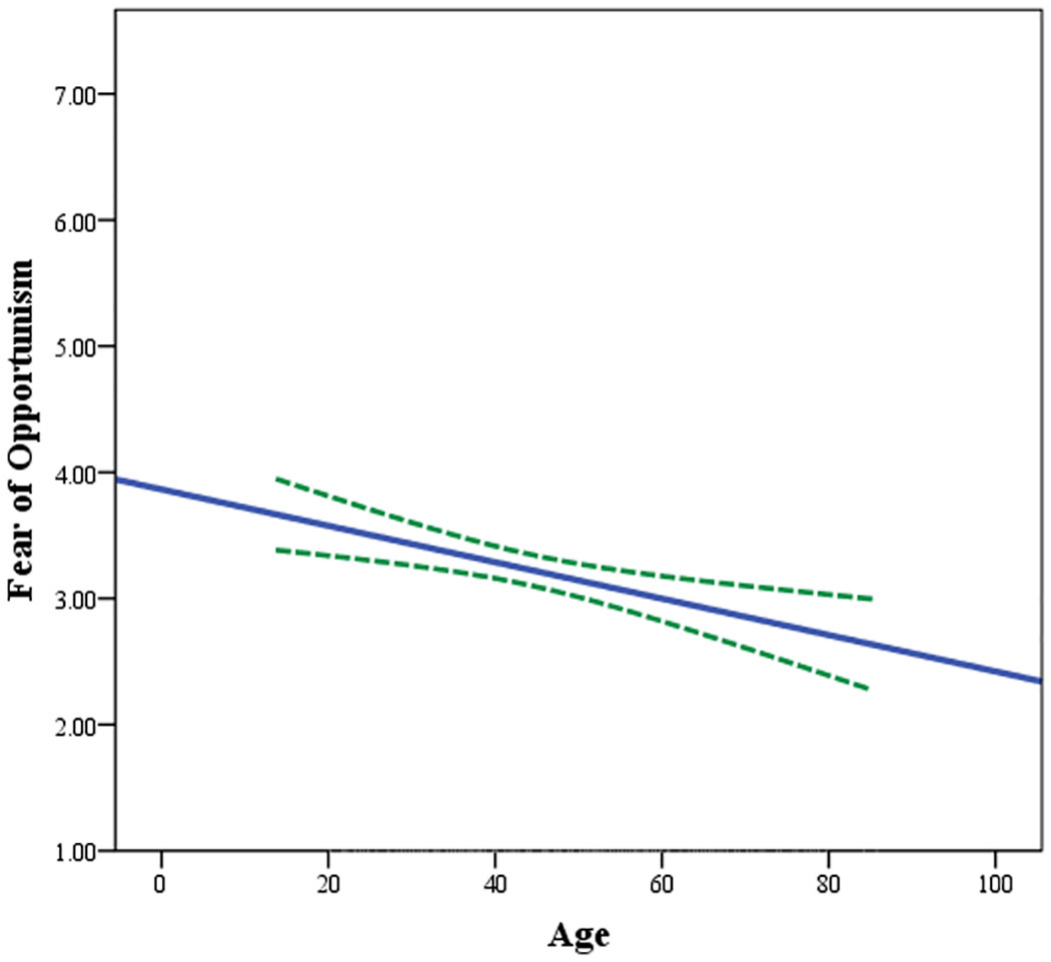
Estimated influence of age on fear of opportunism. The estimation is based on ordinary least squares linear regression. Blue line shows the estimated association between age and fear of opportunism while green lines show the 95% confidence intervals for this estimation.

The association between age and fear of opportunism was negative and statistically significant in all three models (in Model 1 β = -0.15, p < 0.001; in Model 2 β = -0.15, p < 0.001; in Model 3 β = -0.11, p < 0.05). These results suggested that older contest participants experienced less fear of opportunism compared to younger participants. Fig 4 depicts this estimated negative association between age and fear of opportunism.

The education level-fear of opportunism and the income level-fear of opportunism associations were not statistically significant. In Model 1, continuous variables created for measuring education and income level were not significantly associated with fear of opportunism (β = 0.02, p = 0.71 and β = -0.01, p = 0.90, respectively). In Model 2 and 3, none of the dummy variables that were created for measuring education and income levels had a significant association with fear of opportunism (in Model 2 p > 0.30; in Model 3 p > 0.12). These results suggested that fear of opportunism was similar among the contest participants with different education and income levels.

## Discussion

In this paper, we examine fear of opportunism in online global prize-based science contests. Our findings show that contest participants vary in the extent that they experience fear of opportunism. Specifically, we found that fear of opportunism is experienced differently among certain demographic groups: Women and older participants have significantly lower levels of fear of opportunism. One explanation for the significant negative association between age and fear of opportunism is that older people are likely to have better control and regulation of their emotions [23] and therefore would experience lower levels of negative emotions than younger people [24,25]. Another explanation is in line with personality and values research which indicate that, with age, people often become more concerned for others and the broader society and less self-concerned [26,27]. As the main consequence of opportunistic behavior in prize contests is not being able to gain monetary and reputational rewards, people who are less motivated by self-interest of this kind may be less worried about opportunistic behavior and may still be satisfied as long as their knowledge benefits others. Therefore, because older people are less self-concerned, they are likely to have less fear of opportunistic behavior. In terms of the significant association between gender and fear of opportunism, personality studies on gender differences help us explain our finding. Several meta-analyses examining the link between gender and personality traits have found that women are more inclined to believe that people are sincere and have good intentions [28,29]. Therefore, they are less likely to expect opportunistic behavior from other parties, and in turn might be less concerned about their knowledge being used opportunistically. However, these explanations are speculative, as we do not have data on potential mediating constructs suggested above. Future research is needed to determine the exact mechanisms that account for the gender and age differences in the experience of fear of opportunism. For example, researchers might directly measure emotional regulation or general perceptions toward the sincerity of others to test whether these mechanisms actually mediate the link between gender, age and fear of opportunism.

Our study has two main theoretical implications. First, this paper contributes to the recent line of research that investigates the dynamics of prize contests. Prior research contributed to the understanding of prize contests by examining the role of incentives, competition and expertise in such contests [11–14]; however, little research focused on fear of opportunism in prize contests. Our study deepens the knowledge on prize contests by addressing this theoretical gap. Second, our findings question the common assumption in the prior economics literature that fear of opportunism is the same among individuals as we identify a context where this common assumption does not hold. Although we cannot generalize our findings to other contexts with our data, this paper takes the first steps to imply that a more nuanced perspective needs to be taken to comprehensively understand fear of opportunism. We therefore strongly encourage future research to study demographic differences in fear of opportunism in other contexts to develop a more fine-grained theory of knowledge disclosure.

The implications discussed above must be qualified in the light of the limitations of this study. First of all, given the low response rate to our survey, representativeness of our sample is a point of concern. Although we tested and did not find any evidence for non-response bias with our existing data, the possibility of non-response bias cannot be ruled out completely without having data on non-respondents. Unfortunately, the demographic data captured in this study was not available for non-respondents. This common non-response problem, which characterizes surveys that are conducted in online platforms [30], requires our findings to be interpreted with care. Secondly, examining contests in only one online platform may limit the generalizability of our findings. The results presented here are based on an online platform, in which new prize-based science contests are regularly posted and intellectual property rights are legally protected, and potentially idiosyncratic properties of other platforms might influence the results. Although, the platform examined in this study includes a wide range of scientific problems from different disciplines and has large number of participants from all over the world, future research is needed to observe whether our results are generalizable to other platforms for prize-based contests (e.g., those with different intellectual property contracts).

Interestingly, our results showed that, despite experiencing lower fear of opportunism, women are largely underrepresented in prize contests. As noted earlier, such an underrepresentation was also observed in other studies conducted in our context [12]. This finding might raise a concern about the importance of fear of opportunism in determining behavioral outcomes of contest participants; however, the underrepresentation of women in our context might also be a consequence of three main factors. First, overall gender differences in terms of Internet usage might have contributed to this. According to a recent survey, 200 million (or 23%) fewer women than men use the Internet [31]. In Wikipedia, for example, only less than 17% of contributors are women [32]. Secondly, underrepresentation of women in science might also partially explain the lower proportion of women in our sample [33,34]. For instance, percentage of women with a PhD degree in the top 50 physical science departments (i.e., departments with the highest research expenditure) in the United States is less than 25% and this percentage is even lower for faculty members (e.g., about 6.4% for full professors) [34]. Third, prior research found that women are more reluctant in entering competitions [35–37] which could also be another reason why we have less women in our sample. For example, when women and men were given an option to choose between a noncompetitive and a competitive scheme (with the same expected payoff) for completing a task in an experimental setting, only 35% of the women selected the competitive scheme while 73% of the men selected it [35]. Taken together, the Internet and science gender gap, and gender differences in terms of willingness to enter competitions might altogether be important driving forces behind underrepresentation of women in our context. Although beyond the scope of this study, we believe, empirically examining why women are largely underrepresented in prize contests is likely to prove a fruitful avenue for future research.

From a practical standpoint, taking fear of opportunism and the demographic differences in such fears into account in the design of prize contests (e.g., intellectual property protection and compensation structure) and in communication with the participants (e.g., informing them how opportunistic behavior will be avoided) might be important factors in accumulating a large and diversified knowledge pool via prize contests. Our findings on gender and age differences suggest that plans and policies to mitigate the fears of male and younger participants in prize contests are particularly important. Contest organizers therefore might consider taking a proactive approach to inform male and younger participants regarding how the opportunistic behavior will be avoided. Contest organizers can also assess the level of fear of opportunism among contest participants with the scale of this study and, if participants experience high levels of fear of opportunism, they might consider modifying the intellectual property structure or making it more transparent to mitigate such fears. We hope our findings contribute to more effectively harnessing the remarkable potential of prize contests in solving significant scientific problems.

## Supporting Information

S1 Appendix Demographics and Fear of Opportunism Questionnaire. (DOCX)
