# Supplementary material for: Understanding Fear of Opportunism in Global Prize-Based Science Contests: Evidence for Gender and Age Differences
Source: PLoS One. 2015 Jul 31;10(7):e0134898. doi: 10.1371/journal.pone.0134898 (PMC4521938; doi:10.1371/journal.pone.0134898)
Supplement: S1 Appendix — (DOCX) [file pone.0134898.s001.docx]

**S1 Appendix. Demographics and Fear of Opportunism Questionnaire.**

1. What is your gender?

- Male
- Female

2. What was your age at the time of your submission to the challenge?

3. What was your highest academic qualification at the time of your submission to the challenge?

- Less than high school degree
- High school degree or equivalent
- Associate degree or equivalent
- Bachelor’s degree
- Master’s degree
- PhD degree

4. What was your annual net income in US dollars at the time of your submission to the challenge?

- Less than $25,000
- $25,000 to $49,999
- $50,000 to $74,999
- $75,000 to $99,999
- $100,000 to $149,999
- $150,000 to $249,999
- $250,000 or $499,999
- $500,000 or more

5. Please indicate to what extent you agree with the following statements.

|  | Totally Disagree | | Neither agree nor disagree | | | | Totally agree | |  |
| --- | --- | --- | --- | --- | --- | --- | --- | --- | --- |
|  | 1 | 2 | 3 | 4 | 5 | | 6 | 7 |  |
| I think seekers will steal my ideas |  |  |  |  |  | |  |  |  |
| I think seekers will use my solution without paying it. |  |  |  |  |  | |  |  |  |
| I think seeker will change the facts in order not to pay me the award I deserve |  |  |  |  |  | |  |  |  |
| I believe InnoCentive has high integrity |  |  |  |  |  |  | |  | |
| I can expect InnoCentive to treat me in a consistent and predictable fashion. |  |  |  |  |  |  | |  | |
| InnoCentive is not always honest and truthful. |  |  |  |  |  |  | |  | |
| In general, I believe InnoCentive’s motives and intentions are good. |  |  |  |  |  |  | |  | |
| I don’t think InnoCentive treats me fairly. |  |  |  |  |  |  | |  | |
| InnoCentive is open and upfront with me. |  |  |  |  |  |  | |  | |
| I am not sure I fully trust InnoCentive. |  |  |  |  |  |  | |  | |
